# A Thermal Cycler Based on Magnetic Induction Heating and Anti-Freezing Water Cooling for Rapid PCR

**DOI:** 10.3390/mi15121462

**Published:** 2024-11-30

**Authors:** Yaping Xie, Qin Jiang, Chang Chang, Xin Zhao, Haochen Yong, Xingxing Ke, Zhigang Wu

**Affiliations:** 1 State Key Laboratory of Intelligent Manufacturing Equipment and Technology, School of Mechanical Science and Engineering, Huazhong University of Science and Technology, Wuhan 430074, China; yapingx@sansure.com.cn (Y.X.); jiangqin@hust.edu.cn (Q.J.); cchan144@jh.edu (C.C.); xin_z@hust.edu.cn (X.Z.); hcyong@hust.edu.cn (H.Y.); xxke@fzu.edu.cn (X.K.); 2 Sansure Biotech Inc., Changsha 410205, China; 3 Department of Mechanical Engineering, Johns Hopkins University, Baltimore, MD 21218, USA; 4 School of Mechanical Engineering and Automation, Fuzhou University, Fuzhou 350108, China

**Keywords:** thermal cycler, magnetic induction heating, anti-freezing water cooling, rapid heat transfer, polymerase chain reaction

## Abstract

Distinguished by its exceptional sensitivity and specificity, Polymerase Chain Reaction (PCR) is a pivotal technology for pathogen detection. However, traditional PCR instruments that employ thermoelectric cooling (TEC) are often constrained by cost, efficiency, and performance variability resulting from the fluctuations in ambient temperature. Here, we present a thermal cycler that utilizes electromagnetic induction heating at 50 kHz and anti-freezing water cooling with a velocity of 0.06 m/s to facilitate rapid heating and cooling of the PCR reaction chamber, significantly enhancing heat transfer efficiency. A multi-physics theoretical heat transfer model, developed using the digital twin approach, enables precise temperature control through advanced algorithms. Experimental results reveal average heating and cooling rates of 14.92 °C/s and 13.39 °C/s, respectively, significantly exceeding those of conventional methods. Compared to commercial PCR instruments, the proposed system further optimizes cost, efficiency, and practicality. Finally, PCR experiments were successfully performed using cDNA (Hepatitis B virus) at various concentrations.

## 1. Introduction

Infectious diseases caused by pathogenic microbes have long threatened human health and social stability [1,2,3]. Recent coronavirus outbreaks, including SARS in 2003 [4], MERS in 2012 [5], and COVID-19 in 2019 [6], have resulted in substantial suffering and economic disruption globally [7,8,9]. The Polymerase Chain Reaction (PCR) technology is widely recognized as a gold standard for pathogen detection, early diagnosis of infectious diseases, and epidemiological surveillance, due to its unparalleled sensitivity, specificity, and expediency [10,11].

To effectively control the spread of pathogens, rapid detection of results and large-scale screening are essential. Hence, there is an urgent need to enhance the efficiency of PCR and reduce the cost of detection instruments and consumables. The thermal cycler and its associated consumables are critical to increasing efficiency and minimizing PCR system costs. Therefore, many researchers have devoted significant efforts to investigating thermal cycling methods and their corresponding consumables to meet these requirements effectively [12,13,14]. For example, Roche et al. proposed an ultra-fast plasmonic thermal cycler for the PCR that can complete 30 cycles in 54 s [15], but its optical system is complex and large with a precise structure to focus energy. Xu et al. reported a convective thermal cycler without complex temperature control in operation, making it user-friendly for molecular point-of-care testing (POCT) [16], but the reaction chamber consumables’ geometry requires careful consideration, cycle number can’t be directly calculated, and consumables’ shape needs changing for different procedures. While Kopp et al. reported a device for continuous flow PCR systems [17], which utilizes a pump to drive liquid circulation without the need for temperature changes. However, this system faces several challenges, including fixed cycle numbers, reagent adsorption on the microfluidic chip, the presence of bubbles in the microchannels, difficulties in real-time detection, and complexities in the design and production of consumables. Despite the progress made in thermal cycling technology, the high costs of consumables and their usage associated with these methods hinder large-scale screening and limit their commercial viability for widespread adoption.

Compared to the aforementioned thermal cycling technologies, the thermal cycler utilizing thermoelectric cooling (TEC) techniques [18,19] has achieved remarkable success in the market and commercialization. It is majorly attributed to the widespread application and large-scale production of TEC systems, as well as the low cost of consumable tubes and well plates. Notable products currently available, such as Thermo Fisher’s QuantStudio Real-Time PCR Systems [20], Bio-Rad’s CFX Real-Time PCR Systems [21], and Hong-shitech’s SLAN-96P [22], all operate using TEC thermal cycler and have been extensively utilized during the COVID-19 pandemic.

Despite the widespread use of PCR instruments utilizing TEC mode, several challenges persist regarding temperature elevation and reduction. For example, the low thermal transfer efficiency of the TEC in PCR systems can be attributed to the thermal contact resistance between the cold side of the TEC and the PCR reaction chamber, as well as the thermal contact resistance between the hot side and the heat sink. More importantly, the complexity of the TEC’s internal energy conversion system, including the internal thermal contact resistance and the PN junction structure, further contributes to this inefficiency [23]. These factors contribute to insufficient heating and cooling speeds, ultimately failing to meet the growing demand for improved turnaround times (TAT) [24]. Moreover, the heating and cooling capabilities of the Peltier element are affected by variations in ambient temperature [25,26]. Additionally, the relatively high costs [27] associated with Peltier elements hinder the ability to meet market demands for further cost reductions in PCR tests.

To address the limitations of current TEC thermal cyclers used in commercial PCR instruments, this study proposes a solution utilizing magnetic induction heating (MIH) combined with anti-freezing water cooling (AWC). As it minimizes heat waste, reduces heating times, and achieves high power densities without thermal inertia [28,29], MIH has been proven to be more efficient than conventional heating methods. This technique generates eddy currents in the metal target when alternating current flows through an electromagnetic coil, producing heat due to the Joule effect. We utilize this technology to innovate the heating process in PCR thermal cycling, directly heating the PCR chamber. At the same time, water cooling significantly reduces thermal resistance and thus enhances the efficiency of the PCR reaction cycle [30], as the water serves as an effective heat transfer medium, utilizing forced convection to cool the PCR chamber rapidly. By introducing antifreeze to the cooling water, AWC prevents freezing, ensuring that the cooling process remains efficient and that optimal performance of the PCR reaction cycle is maintained even under low-temperature conditions. To validate the new cycler, we integrated an optical module, a control module, and the thermal cycler itself to form a qPCR system. Finally, a quantitative PCR (qPCR) is conducted with cDNA from the Hepatitis B virus. The results demonstrate the enhanced performance of the new thermal cycling approach, highlighting its potential advantages over traditional TEC systems.

## 2. Principles and the Overall Design

**Figure 1:**
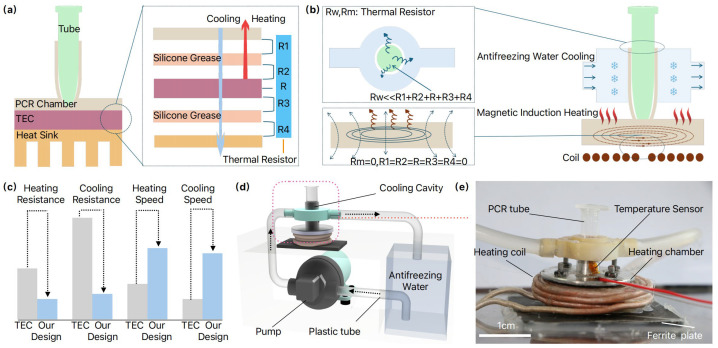
Illustration of MIH and AWC-based thermal cycler. (**a**) Schematic diagram depicting the heat resistance of conventional thermoelectric (TEC) systems, $R_{1}$ is the contact thermal resistor between the PCR chamber and Silicone Grease, $R_{2}$ is the contact thermal resistor between Silicone Grease and Peltier’s upper surface, R is the complex thermal resistor of TEC, $R_{3}$ is the contact thermal resistor between Peltier’s lower surface and Silicone Grease, and $R_{4}$ is the contact thermal resistor between Silicone Grease and Heat sink; (**b**) Schematic diagram illustrating the heat resistance characteristics of our proposed design, $R_{w}$ is the complex thermal resistor of AWC, $R_{m}$ is the thermal resistor of MIH; (**c**) Performance comparison plots between the TEC and ours; (**d**) 3D conceptual representation of the proposal cycler; (**e**) Photograph of the thermal cycler. Mineral oil is added into the PCR tube to prevent water evaporation, and temperature is monitored in real-time by a PT100 sensor and regulated using a proportional-integral-derivative (PID) control algorithm.

The slow heating and cooling rates of the TEC-based thermal cycler are primarily attributed to its high thermal resistance and thermal inertia, which result in low heat transfer efficiency. Therefore, it is important to analyze the detailed thermal resistance and thermal inertia of the TEC-based thermal cycler and figure out their influence on the system. As in Figure 1a, traditional TEC thermal cyclers operate based on the Peltier effect, utilizing thermal silicone grease to bond the PCR reaction chamber to the heat sink for thermal cycling. Between the PCR reaction chamber and the thermal silicone grease, a thermal contact resistance $R_{1}$ is formed. Similarly, a contact thermal resistance $R_{2}$ occurs between the thermal silicone grease and the upper surface of the Peltier element. The thermal resistance of the Peltier element itself is designated as R [23], while $R_{3}$ represents the thermal contact resistance between the lower surface of the Peltier and the thermal silicone grease. Finally, the thermal contact resistance between the thermal grease and the TEC heat sink is denoted as $R_{4}$. We will focus on reducing or eliminating the aforementioned thermal resistances to improve thermal efficiency, thereby enabling faster heating and cooling rates.

**Figure 2:**
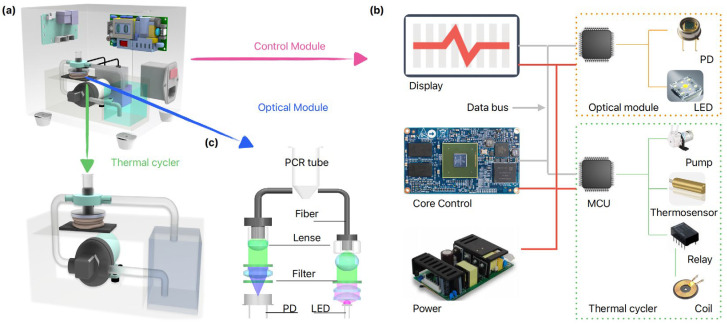
Illustration of the MIH and AWC-based qPCR device. (**a**) 3D conceptual diagram of the qPCR device; (**b**) Schematic diagram of the Control module; (**c**) Schematic diagram of the Optical detection module.

As illustrated in Figure 1b, for the cooling process, we developed an AWC model by simplifying the thermal resistance network, eliminating the combined thermal resistances $R_{1}$, $R_{2}$, R, $R_{3}$, $R_{4}$ associated with the TEC thermal cycler. Instead, the model incorporates only the thermal resistances of the cooling water and the reaction chamber. By employing a continuous flow of anti-freezing cooling water to remove heat in real-time, the heat exchange efficiency is significantly enhanced, leading to a substantial reduction in the overall thermal resistance, denoted as $R_{w}$. At the same time, we developed an MIH model to address the thermal resistance issues during the heating process. When alternating current flows through the electromagnetic coil, eddy currents are generated at the metal bottom of the PCR reaction chamber. These eddy currents produce heat due to the Joule effect, which raises the temperature of the PCR chamber, thus providing direct heating. This effectively eliminates the thermal resistances in the TEC thermal cycler, resulting in $R_{1}$, $R_{2}$, R, $R_{3}$, $R_{4}$ and an overall thermal resistance $R_{m}$ of zero. Compared to conventional TEC thermal cyclers, our design exhibits significantly reduced thermal resistance, enhanced heat transfer efficiency, and improved rates of temperature rise and fall, Figure 1c. We integrate the MIH and AWC modules in our thermal cycler design, with an overview schematics in Figure 1d and a physical representation in Figure 1e. To evaluate the performance of the thermal cycler, we developed a quantitative PCR (qPCR) setup that incorporates an optical module and a control module, as depicted in Figure 2.

## 3. Materials and Methods

### 3.1. Detailed Thermal Cycler Design

Regarding to the MIH module, an electromagnetic coil (20 turns) is affixed to the upper side of a magnetic isolation sheet using glass adhesive. The alternating current passing through the electromagnetic coil induces eddy currents in the metal sheet adhered to its upper surface, resulting in heat generation. This heat is then transferred to the thermal chamber to elevate the temperature of the PCR reaction system contained within a 0.2 mL PCR tube (Figure 1e). For the AWC building, a pump (XDHY, purchased from Taobao, China) circulates anti-freezing water through a plastic tube. The water then flows through a cooling cavity, fabricated via 3D printing, that surrounds the heating chamber. This process effectively lowers the temperature of the heating chamber and cools the PCR reaction system contained within a PCR tube (Figure 1e). To facilitate the attachment of a temperature sensor to the surface of the heating chamber (Figure 1e) and also accommodate the placement of optical fibers for reaction detection (Figure 2a), a cooling cavity was designed to partially surround the heating chamber.

### 3.2. Integrated qPCR Device

The qPCR device consists of the aforementioned thermal cycler (Figure 1e), as well as the core control module (Figure 2b) and optical model (Figure 2c). The subsystem of the qPCR device is introduced thoroughly in Supplementary Materials Sections S.1–S.4.

### 3.3. Amplification Program

The PCR amplification program was established as follows: (1) UNG enzyme reaction at 50 °C for 2 min; (2) Taq enzyme activation at 94 °C for 5 min; (3) 45 cycles consisting of: denaturation at 94 °C for 15 s, followed by annealing/extension at 57 °C for 30 s.

### 3.4. PCR Reagents

A hepatitis B virus (HBV) one-step PCR reagent kit (Catalog No. 24009-2) was obtained from Sansure Biotech Co., Ltd. (Changsha, China). Each test requires 15 µL of reagent. This kit is designed for the detection of HBV-specific polynucleotides using TaqMan fluorescent probe-based PCR technology. The probe HBV-P (SEQ No. 1) is derived from the conserved region of the HBV surface antigen S gene and is used in conjunction with upstream and downstream primers, also from this conserved region, for amplification. The upstream primer, HBV-F (SEQ No. 2), and the downstream primer, HBV-R (SEQ No. 3), are used in the amplification process.

SEQ No.1: 5′-CCTCTTCATCCTGCTGCTATGCCTCATCTTCTTATTGG-3′;

SEQ No.2: 5′-ATCGCTGGATGTGTCTGCTGCGTTTT-3′;

SEQ No.3: 5′-CTGGAATTAGAGGACAAACGGGCAACAT-3′.

The HBV quantitative reference material is derived from the HBV DNA national standard (purchased from the Chinese National Institute for the Control of Pharmaceutical and Biological Products), and calibrated using highly positive HBV serum, which is then diluted with HBV-negative serum.

### 3.5. FEA Simulation

Multi-physics coupling simulations were performed using COMSOL 5.6a (COMSOL Inc., Stockholm, Sweden). An induction heating model was employed to simulate the heating process of the PCR chamber. Various design parameters, including alternating current frequency, number of coil turns, clearance distance between the coil and the PCR chamber, and current value were adjusted to identify the optimal configuration. Additionally, fluid-solid coupling and convection heat transfer modules were utilized to simulate the cooling processes, with the temperature of the cooling fluid set to 5 °C.

## 4. Results and Discussion

### 4.1. MIH Process Design and Simulation Analysis

**Figure 3:**
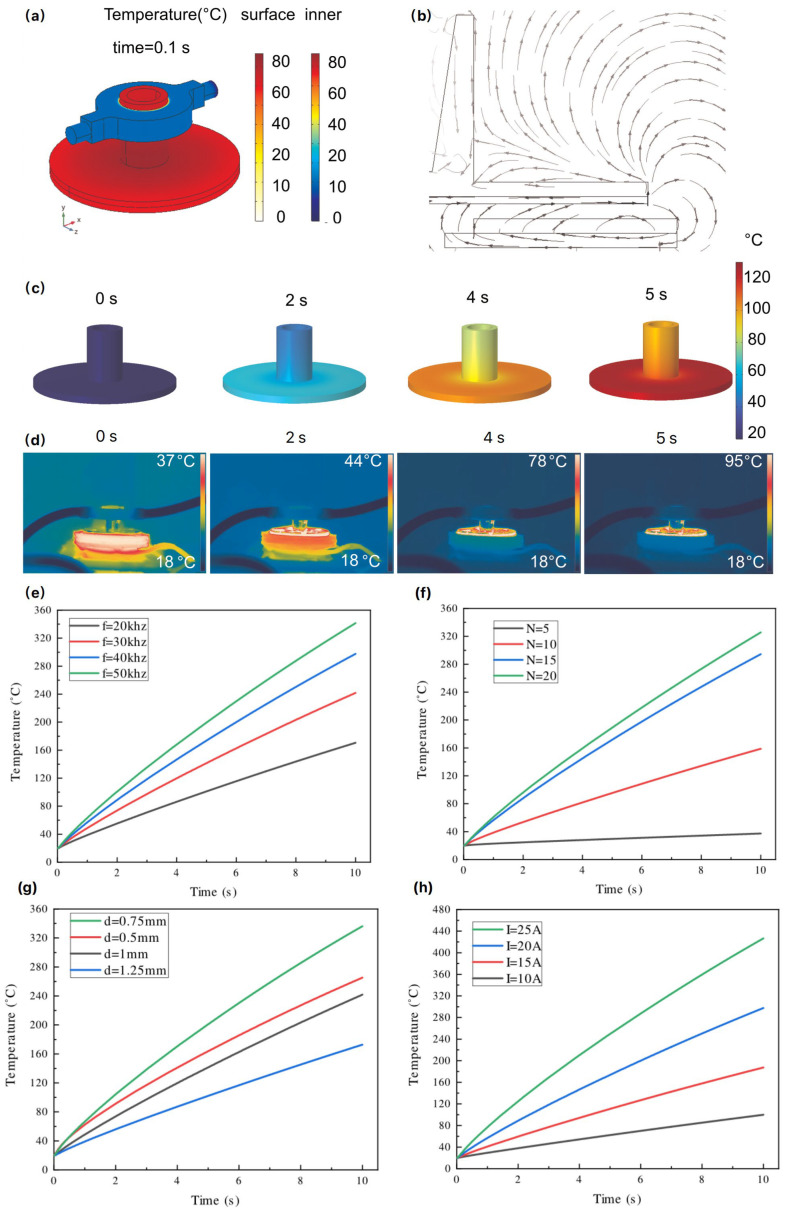
Simulation and experimental results during the magnetic induction heating. (**a**) FEA model of the PCR control device; (**b**) Distribution of flux density of the induced magnetic field; (**c**) FEA results showing temperature variation during induction heating; (**d**) Thermography illustrating the PCR chamber induction heating process; (**e**) Relationship between different excitation frequencies and temperature; (**f**) Impact of coil turns on temperature; (**g**) Effect of the gap between the PCR chamber and coil on temperature; (**h**) Influence of electrical current on temperature.

The use of the MIH method to heat the PCR chamber is theoretically viable [31], Figure 3a. We have also developed a finite element analysis (FEA) model based on Figure 3b to investigate and optimize the MIH design. The simulation of the MIH process revealed that the bottom of the PCR chamber warmed first (Figure 3c), with heat subsequently transferring from the bottom to the body of the chamber. Although a temperature gradient exists throughout the chamber during rapid heating, the temperature of the DNA reaction solution can be regulated by the controlling algorithm. The thermographic images of the induction-heated PCR chamber corroborate the heat transfer process observed in our simulations (Figure 3d). Figure 3c,d record the simulation temperatures and thermographic data of the reaction chamber at various time points (2 s, 4 s, and 5 s). It is evidenced that the temperature distribution in both the simulated and thermographic reaction chambers improves over time, and MIH can effectively achieve the temperatures required for the PCR chamber reactions.

To enhance the heating rate, it is essential to identify the optimal parameters. Based on the physical model shown in Figure 3b, which illustrates the spatial magnetic field distribution of MIH heating, the heating power generated by eddy currents can be derived using Maxwell’s equations and referenced literature [31], as illustrated in Equations (1) and (2).

(1) $$P_{D}=\frac{\pi }{2\sigma }\int_{0}^{\infty }({J_{s}(r))}^{2}\delta rdr,$$

(2) $$J_{s}\left(r\right)=\frac{j\omega \mu_{0}\sigma NI_{m}a}{2}\int_{0}^{\infty }J_{1}\left(ka\right)J_{1}\left(kr\right)e^{-kz\;}dk$$

where j is an imaginary unit, ω is the exciting frequency (rad/s), $\mu_{0}$ is permeability of the free space. Thickness δ is the skin depth in the disk with electrical conductivity σ, a is a coil of radius, N is coil turns and a coil carrying magnetizing current of amplitude $I_{m}$. $J_{1}(kr)$ is the Bessel Function of order 1 and argument kr, small k approaches zero and large k denominate approaches unity, r, z are the radial and axial coordinates with the origin at the coil center.

According to the key factors outlined in Equations (1) and (2), none of the parameters are dependent on ambient temperature, indicating that MIH power is independent of environmental conditions. This feature resolves the issue of fluctuating heating efficiency seen in TEC systems, which can be influenced by changes in ambient temperature. While a PID algorithm in TEC systems can compensate for temperature variations, such fluctuations still impact the performance and optimal operating conditions of the TEC [25,26]. In contrast, the MIH remains unaffected from such changes. Additionally, a simulation was conducted using finite element methods (FEM) in COMSOL Multi-physics, aiming to improve the MIH efficiency and performance of the PCR system by carefully adjusting the key factors in the aforementioned equations. To accommodate the dimensions of the PCR reaction chamber, we selected a coil with a fixed radius and focused our optimization efforts on a range of parameters: MIH frequencies from 20 kHz to 50 kHz, coil turns ranging from 5 to 20, and driving currents adjustable between 10 A and 25 A. We also considered the clearance distance between the coil and the PCR chamber varying from 0.75 mm to 1.25 mm. According to Figure 3e–h, increasing the number of coil turns, MIH current, MIH frequency, and decreasing clearance distance all contribute to greater heating power of the eddy currents in the disc, thereby accelerating the rate of temperature rise. Ultimately, in this design, we selected a coil with a radius of 2 cm, a clearance distance of 0.75 mm, a current of 15 A, and an excitation frequency of 50 kHz for MIH.

### 4.2. AWC Process Design and Simulation Analysis

**Figure 4:**
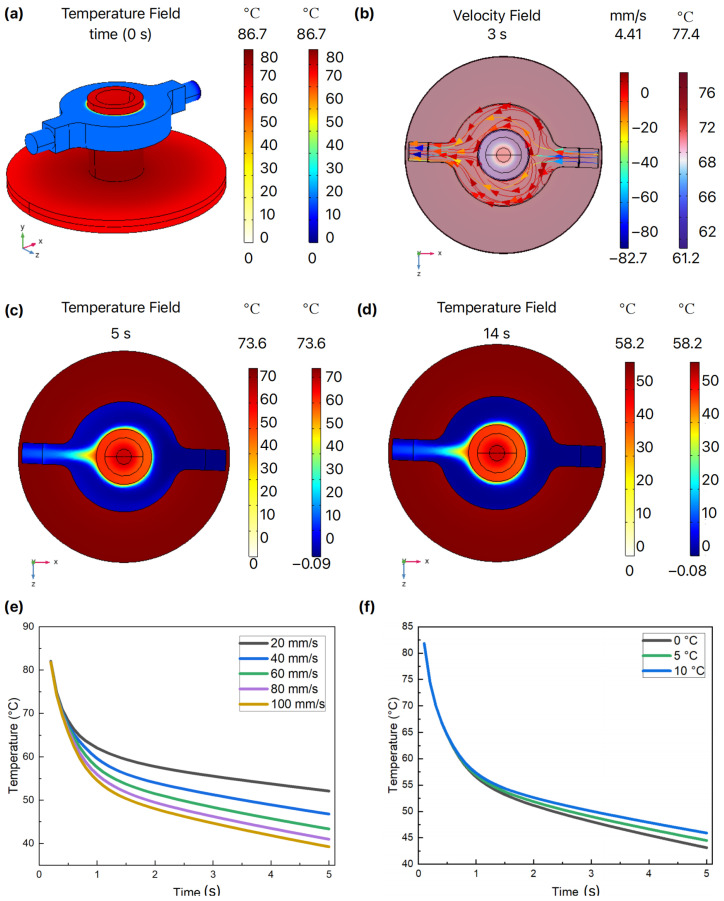
COMSOL cooling simulation results. (**a**) Initial cooling state; (**b**) Temperature of velocity field distribution at 3 s; (**c**) Fluid temperature field at 5 s; (**d**) Temperature of velocity field distribution at 14 s; (**e**) Effect of water velocity on temperature; (**f**) Relationship between the initial cooling water temperature and chamber temperature.

We have also developed an FEA model to investigate and optimize the AWC design. The simulation results show that as the cooling water circulates, the temperature of the PCR chamber gradually decreases. The temperature distribution and fluid velocity field were recorded at 3 s, 5 s, and 14 s intervals, along with the overall temperature field distribution. Figure 4a–d illustrate how the temperature of the reaction chamber decreased over time, eventually stabilizing at the target value. To further enhance the cooling rate, identifying the optimal parameters is crucial. The core principle of AWC is based on convective cooling. We further examine several critical design parameters related to convection cooling. Based on the physical model established in Figure 1e and following Newton’s law of cooling, we can express this relationship as shown in Equation (3).

(3) $$\emptyset =\alpha A\left(∆T\right),$$

where, heat flow ∅ (W), heat transfer area $A\;(m^{2})$, temperature difference ∆T (°C), and convective heat transfer coefficient α. As indicated by Equation (3), a faster cooling rate during PCR requires the removal of a greater amount of heat per unit of time. The heat carried away by the flowing liquid can be approximated by the Formula (4).

(4) $$P_{c}=C_{\rho }\rho vS{(T}_{out}-T_{in}),$$

where $P_{c}$ is the thermal power, $C_{\rho }$ is the specific heat capacity, ρ is the liquid density, v is the fluid flow velocity, S is the cross-section area of the pipeline, $T_{in}$ is the temperature of the liquid entering the cooling zone, and $T_{out}$ is the temperature of the liquid exiting the cooling zone.

According to Equation (4), the AWC system relies on the continuous flow of cooling water, which facilitates real-time heat removal. This flow ensures that the reaction chamber is constantly surrounded by cooling water, thereby reducing the system’s thermal resistance and enhancing its thermal conductivity. Consequently, the temperature within the PCR chamber decreases rapidly. To ensure the low-temperature characteristics of the cooling water, we designed a water tank with a volume of 288 mL (Figure 1d) and dimensions of 80 mm × 60 mm × 60 mm, which was mounted on a TEC patch to facilitate water cooling. A PID control algorithm was employed to maintain a stable temperature in the water tank. To maintain a stable flow under low-temperature conditions, antifreeze is incorporated into the cooling water. Based on the above, the liquid flow velocity and the temperature of the incoming liquid are critical parameters affecting heat transfer efficiency.

After determining the pipe diameter and material, we set up a simulation scenario with an initial heating chamber temperature of 95 °C. Cooling water was introduced at temperatures ranging from 0 °C to 10 °C, with flow velocities between 20 mm/s and 100 mm/s. As shown in Figure 4e, higher water velocities enhanced the cooling rate, but this effect plateaued at flow velocities above 60 mm/s. Given the mechanical limitations of the water pump, a flow velocity of 60 mm/s was selected as the optimal parameter. Additionally, Figure 4f demonstrates that cooling efficiency improved as the initial temperature of the cooling water decreased from 10 °C to 0 °C. However, after 5 s of cooling, the final temperature varied by only about 2 °C within this range, indicating that the initial temperature of the cooling water had a minimal impact on chamber cooling. Based on these findings, cooling water with an initial temperature of 5 °C and a flow velocity of 60 mm/s was chosen. Under these conditions, it has been calculated that a maximum cooling rate of approximately 12 °C/s can be achieved as in Figure 4e, which hints that AWC is a highly efficient in cooling.

### 4.3. Temperature Controlling Performance

**Figure 5:**
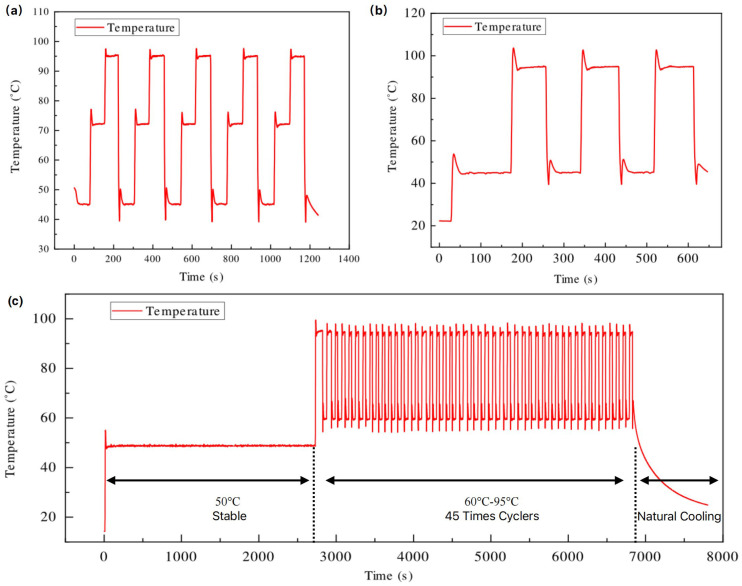
Temperature curves: (**a**) Three setpoints (45–72–95 °C); (**b**) Two setpoints (45–95 °C); (**c**) A complete PCR controlling program.

Following the simulations of MIH and AWC, we developed a 3D design based on the obtained parameters and fabricated a physical prototype of the thermal cycler. Then we conducted a series of cycling experiments to evaluate the temperature control properties of the prototype. As shown in Figure 5a, a cycling test was performed with three setpoints (45 °C, 72 °C, and 95 °C) to assess the stabilization and accuracy of temperature control. During the temperature rise, an overshoot was observed, which can be suppressed through the PID algorithm. Also, the temperature overshooting is intentionally introduced to improve the heating rate and the heat transfer to the solution. The maximum temperature fluctuation can be calculated using Equation (5).

(5) $$T_{f}=T_{max}-T_{min},$$

where $T_{max}$ and $T_{min}$ represent the maximum and minimum temperatures, respectively, after the temperature has stabilized at the setpoint for 10 s. As detailed in Table 1, the average $T_{f}$ fluctuation of the proposed thermal cycler can be maintained within 0.2 °C for each setpoint, indicating excellent stabilization and repeatability.

The temperature changing rate of the PCR chamber is also investigated by conducting a cycling test between two setpoints (50 °C and 90 °C), as illustrated in Figure 5b. The heating and cooling rate ($T_{v}$) can be calculated using Equation (6).

(6) $$T_{v}=\left|\frac{T_{a}-T_{b}}{t}\right|,$$

where $T_{a}$ and $T_{b}$ are the temperatures that are very close to 50 °C and 90 °C, respectively. Therefore, the maximum heating and cooling rate can reach 20.29°C/s and 21.34 °C/s, while the average heating and cooling rate can reach 14.92 °C/s and 13.39 °C/s (Table 1). This demonstrates that the thermal cycler based on MIH and AWC achieves a faster temperature rate compared to other related works described in the previous introduction.

Moreover, the temperature control accuracy ($T_{\alpha }$) of the proposal thermal cycler can be calculated based on the differences between the mean temperature and the setpoint temperature. The mean temperature is determined as the average of five measurements taken over a continuous 10-s interval. Table 1 presents the results for the temperature control accuracy ($T_{\alpha }$), showing values of 0.07 °C, 0.19 °C, and 0.13 °C at different setpoint temperatures. Regarding the heating and cooling performance of the thermal cycling process, a comparative analysis was conducted with commercial PCR instruments, which demonstrated a significant advantage (Table 1).

Based on the experimental results of the thermal cycler, the temperature control accuracy, precision, and heating/cooling rates demonstrate its feasibility for rapid PCR applications. So, a comprehensive PCR control program was established to demonstrate the practical applications of our instrument (Figure 5c). 

The complete PCR process consists of three stages, including maintaining a stable temperature at 50 °C, performing 45 cycles of temperature cycling between 57 °C and 94 °C, and allowing for natural cooling finally. Figure 5c shows that the temperature ramping curves throughout the entire PCR process align with the expectations of our design. Due to the presence of plastic PCR tubes between the heating chamber and the solution, a temperature deviation is observed. Measurements show that at 50 °C, the solution temperature is 0.50 °C lower than the chamber temperature, while at 94 °C, it is 1.77 °C lower. However, at 57 °C, the solution temperature exceeds the chamber temperature by 1.20 °C (see Supplementary Materials Table S7). This deviation will be further validated through actual PCR experiments to assess its impact.

### 4.4. qPCR Verification

To validate the performance of the thermal cycler, we compared it with commercially available PCR products. PCR amplification experiments were conducted using both our designed device with the proposed thermal cycler and the SLAN 96P PCR instrument.

With the HBV one-step reagent, we prepared quantitative reference materials *A* (4 × 10^7^ IU/mL), *B* (4 × 10^6^ IU/mL), *C* (4 × 10^5^ IU/mL), *D* (4 × 10^4^ IU/mL), *E* (diluted 10 times from *D*, 4 × 10^3^ IU/mL), as well as the limit of detection (LOD) at 30 IU/mL and a no-template control (NTC), each placed in individual PCR tubes (0.2 mL). The samples *A*, *B*, *C*, *D*, *E*, and LOD were tested in triplicate, while the NTC was tested once. The $C_{t}$ values obtained from the proposed qPCR device and the log values of the concentrations of the quantitative reference materials were used for linear fitting and compared with the fitting curves and correlation coefficients obtained from the commercial instruments.

**Figure 6:**
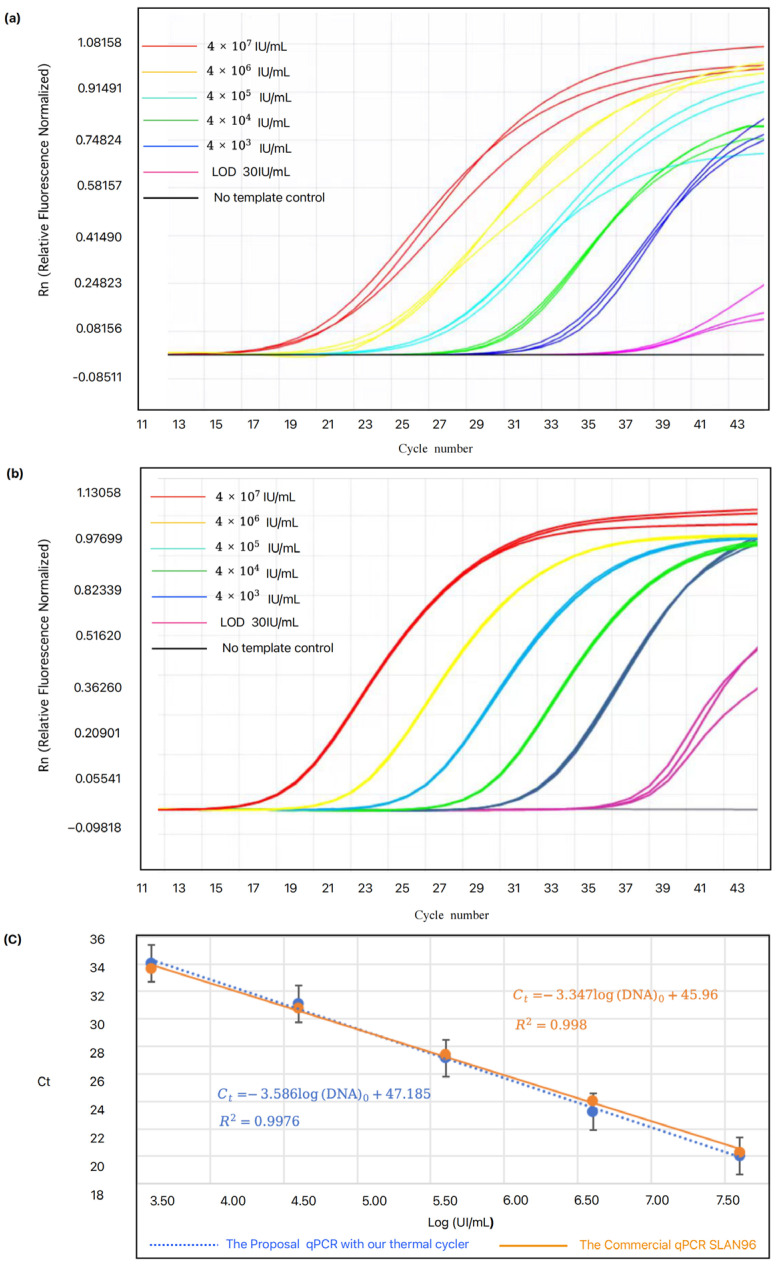
PCR Test Performance. (**a**) Testing different concentrations using the proposed PCR device; (**b**) Testing different concentrations using the commercial PCR device; (**c**) Linearity assessment of the proposed PCR device compared with the commercial PCR device.

The results indicate that all internal standards were successfully detected, and there was no contamination in the NTC. The fluorescence intensity trends corresponding to each cycle for different concentration ranges are depicted in Figure 6a (the qPCR device with the proposal thermal cycler) and Figure 6b (the commercial SLAN 96P qPCR instrument). The average $C_{t}$ values obtained from our designed thermal cycler for concentration *A* is 20.11, for concentration *B* is 22.97, for concentration *C* is 27.3, for concentration *D* is 31.3, and for concentration *E* is 33.68. In comparison, the average $C_{t}$ values from the commercial SLAN 96P are 20.22 for concentration *A*, 23.98 for concentration *B*, 27.37 for concentration *C*, 30.76 for concentration *D*, and 33.66 for concentration *E*. The absolute differences in $C_{t}$ values across the various concentrations were 0.12, 1.01, 0.07, 0.54, and 0.02, indicating a close agreement with the SLAN 96P. Notably, the thermal cycler based on MIH and AWC achieved a detection time of only 65 min, which is 16 min faster than those of the SLAN 96P. This suggests both efficiency and accuracy in the detection process. Figure 6a,b also present the fluorescence curves at LOD for the HBV reagent. Our device with the thermal cycler successfully detected the LOD of HBV at 30 IU/mL, which is comparable to commercial PCR instruments, with an absolute difference in $C_{t}$ values of only 0.44, demonstrating a satisfactory sensitivity.

Figure 6c shows the standard curves extracted from Figure 6a,b, where the $C_{t}$ value is plotted as a function of cDNA concentration. Both the designed devices with our thermal cycler and the commercial SLAN 96P employed five-point linear fitting, yielding a correlation coefficient ($R^{2}$) of 0.9976 for our device and 0.998 for the SLAN 96P (Figure 6c). The performance of both systems is comparable, and the linear ranges meet the established requirements for quantification. Although there is temperature deviation between the heating chamber and reaction solution, the PCR amplification process was not significantly impacted. The above results confirm that our thermal cycler is suitable for rapid PCR systems, demonstrating excellent performance. Although the designed thermal cycler demonstrates excellent performance in nucleic acid testing, there is still room for further optimization. For example, adding a melt curve analysis function could enhance the system’s multiplex target detection capability. We also observed potential differences in temperature distribution within the PCR solution, which could be further investigated to enhance PCR amplification efficiency and performance in future studies.

## 5. Conclusions

We designed a new thermal cycler based on MIH and AWC for rapid PCR. This technology achieves approximately 100% and 150% increases in average heating and cooling rates, respectively, with the rates reaching 14.92 °C/s. Furthermore, the faster heating and cooling rates would significantly enhance the overall amplification process, especially for amplifying small DNA fragments (e.g., 150 bp), allowing for shorter durations of each PCR step [32]. The proposed thermal cycler utilizes low-cost components such as electronics, pumps, and plastic tubes, resulting in a production cost of approximately $70. This represents a substantial cost reduction compared to traditional commercial TEC solutions. We evaluated the performance of the proposed thermal cycler integrated into our qPCR device by amplifying cDNA from the Hepatitis B virus. The results confirm its capability for quantitative detection, demonstrating that the overall PCR reaction time is reduced by 20% while maintaining the necessary PCR cycle times. The performance of our device is comparable to that of widely used commercial PCR instruments. This heating and cooling system not only is suitable for traditional commercial PCR instruments but also holds potential for development in rapid POCT applications.

## Figures and Tables

**Table 1:** Performance comparison between our system and other commercial instruments. (Detailed test results of our design are introduced in Supplementary Materials Section S.5; ‘-’ denotes data absence).

		Our Design	SLAN96 [22]	CFX96 [21]	Qs5 [20]
Heating	Avg ramp rate	14.92 °C/s	-	3.3 °C/s	-
	Max ramp rate	20.29 °C/s	4.0 °C/s	5 °C/s	6.5 °C/s
Cooling	Avg ramp rate	13.39 °C/s	-	3.3 °C/s	-
	Max ramp rate	21.34 °C/s	4.0 °C/s	5 °C/s	6.5 °C/s
Accuracy	45 °C	0.20	0.10	0.20	0.25
	72 °C	0.20	0.10	0.20	0.25
	95 °C	0.2	0.10	0.20	0.25
Precision	45 °C	0.07	0.10	0.20	0.25
	72 °C	0.19	0.10	0.20	0.25
	95 °C	0.13	0.10	0.20	0.25

## Data Availability

Raw data are available upon request.

## Supplementary Materials

The following supporting information can be downloaded at: https://www.mdpi.com/article/10.3390/mi15121462/s1, Section S.1: Core Control Module; Section S.2: Optical Module; Section S.3: ZVS circuit; Section S.4: ZVS Thermal cycler Control system structure; Section S.5: Detailed test results for Thermal cycler Control system; Figure S1: Schematic of the ZVS circuit; Figure S2: Schematic of thermal cycler control system structure; Table S1: Precision of Temperature Control; Table S2: Average heating rate test results; Table S3: Average cooling rate test results; Table S4: Maximum heating rate test results; Table S5: Maximum cooling rate test results; Table S6: Accuracy of Temperature Control; Table S7: Temperature deviation between the PCR chamber and the reaction solution.
