# Supplementary material for: A Thermal Cycler Based on Magnetic Induction Heating and Anti-Freezing Water Cooling for Rapid PCR
Source: Micromachines (Basel). 2024 Nov 30;15(12):1462. doi: 10.3390/mi15121462 (PMC11679298; doi:10.3390/mi15121462)
Supplement: Supplementary file 1 [file micromachines-15-01462-s001.zip › micromachines-3343613-supplementary.pdf]

Supporting Information

S.1. Core Control Module

The core control microcontroller (MCU, NXP i.MX6Q) communicates with the optical detection sub-module MCU (STM32F103) via the RS485 protocol to generate excitation light. Simultaneously, fluorescence signals detected by a photodiode (PD) are captured by an analog-to-digital converter. The core MCU also interfaces with the thermal cycling sub-module MCU over RS485 to drive the electromagnetic coil for heating and control the pump for anti-freezing water circulation, thereby enabling cooling. Temperature is monitored in real-time by a PT100 sensor and regulated using a proportional-integral-derivative (PID) control algorithm. Finally, the core MCU communicates with the display sub-module via I2C and LVDS interfaces to manage the content display.

S.2. Optical Module

The optical detection system consists of six optical detection channels, which operate on similar principles. Taking the ROX channel as an example, excitation light is generated by a surface-mounted LED (wavelength: 580-590 nm, model T3535Y1CT, PARA LIGHT, Taiwan). The excitation light first passes through a glass lens (CHIOPT Co., LTD, China) for collimation, then through a filter with a central wavelength of 580 nm (20 nm OD6, Beijing Bodina Optical Co., LTD, China). After focusing by a glass spherical lens (CHIOPT Co., LTD, China), the excitation light is directed into an excitation light-conducting optical fiber (diameter: φ1.4 mm, Nanjing Chunhui Science & Technology Industrial Co., Ltd.) and transmitted to the sample.

The emitted fluorescence is generated when the sample is illuminated by excitation light and transmits through a fluorescence-receiving glass optical fiber positioned at a 90-degree angle to the excitation optical fiber. The fluorescence first passes through a meniscus glass lens (CHIOPT Co., LTD, China) for collimation, followed by a symmetrical bi-convex lens (CHIOPT Co., LTD, China) for focusing. Finally, the fluorescence passes through a bandpass filter (central wavelength: 611 nm, 14 nm OD6, Beijing Bodina Optical Co., LTD, China) before reaching the silicon photodiode (S2386-44K, Hamamatsu), where it is converted into an electrical signal.

S.3. ZVS circuit

A zero-voltage switching (ZVS) oscillation circuit is used to drive the coil and generate an electromagnetic field. In this circuit, the MOSFETs operate in a zero-voltage state, minimizing switching losses and improving efficiency. Upon powering the circuit, a positive voltage is applied to the gates of both MOSFETs. Due to component variations, one MOSFET (Q1) turns on before the other (Q2). When Q1 is activated, current flows through L1 and Q1, which causes Q2 to turn off as its gate current decreases. Diode D1 ensures that Q2 remains off. As capacitors C1 and C2 charge simultaneously, they form an LC oscillation circuit with inductors L1 and L2. Once fully charged, C1 and C2 discharge, causing the source voltage of Q2 to drop to zero, at which point Q1 is turned off by diode D2. This reverses the charging direction of C1 and C2, perpetuating the oscillation process (Figure S1).

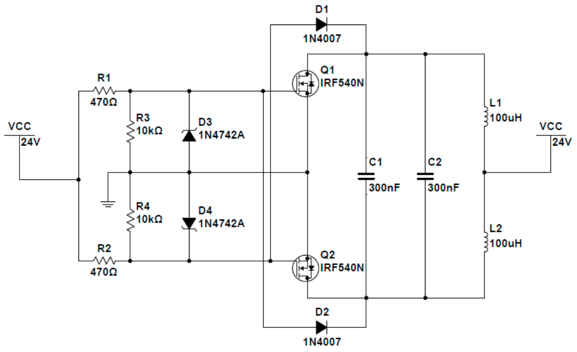

Figure S1. Schematic of the ZVS circuit.

S.4. ZVS Thermal cycler Control system structure.

The thermal cycler control system consists of three main control units and a feedback unit, with a PID algorithm for precise temperature regulation. The first unit, the MIH unit, includes solid-state relays, ZVS circuits, and MIH coils that generate heat via electromagnetic induction. The second unit, the AWC unit, controls the pump for cooling, while the third unit manages auxiliary temperature control through a fan. The auxiliary cooling module was employed to mitigate the temperature overshooting of the PCR tube. The feedback unit comprises sensors and a data acquisition transmission circuit, enabling real-time monitoring and adjustments. The operation of these units is regulated based on feedback inputs, ensuring optimal performance and stability (Figure S2).

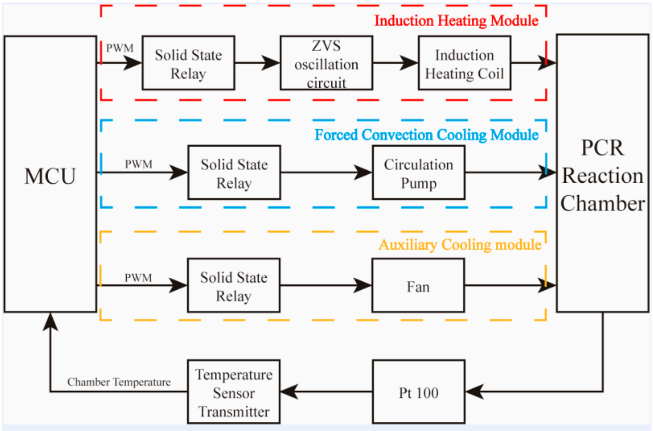

Figure S2. Schematic of thermal cycler control system structure.

S.5. Detailed test results for Thermal cycler Control system

Table 1 is the test results summary of Table S1, S2, S3, S4, S5, and S6, and the details are as follows.

Table S1. Precision of Temperature Control.

Setpoints		45 (°C)	72 (°C)	95 (°C)
	T_{max}	45.25	72.30	95.22
cycle 1	T_{min}	44.92	71.90	94.82
	$Diff = \Delta T/2$	0.16	0.20	0.20
	T_{max}	45.28	72.27	95.21
cycle 2	T_{min}	44.88	71.86	94.83
	$Diff = \Delta T/2$	0.20	0.20	0.19
	T_{max}	45.26	72.27	95.20
cycle 3	T_{min}	44.91	71.86	94.85
	$Diff = \Delta T/2$	0.17	0.20	0.18
cycle 4	T_{max}	45.33	72.32	95.15
	T_{min}	44.95	71.92	94.74
	$Diff = \Delta T/2$	0.19	0.20	0.20

cycle 5	T_{max}	45.25	72.20	95.12
	T_{min}	44.84	71.81	94.71
	$Diff = \Delta T/2$	0.20	0.19	0.20
Maximum temperature fluctuation (T_f) °C		0.20	0.20	0.20

Table S2. Average heating rate test results

	T_a(°C)	T_b(°C)	t	v (°C /s)
Cycle 1	49.97	88.44	2.512	15.32
Cycle2	49.89	87.83	2.513	15.10
Cycle 3	48.34	89.61	2.875	14.36
Mean Average heating rate (°C/s)			14.92	

Table S3. Average heating rate test results

	T_a(°C)	T_b(°C)	t	v (°C /s)
Cycle 1	93.82	49.17	3.29	13.59
Cycle2	88.65	49.63	2.88	13.57
Cycle 3	88.02	50.02	2.92	13.01
Mean Average cooling rate (°C/s)			13.39	

Table S4. Maximum heating rate test results

**Time**	**Real-time temperature （°C）**	**Δt Temperature change value**	**v (°C /s)**
0s	49.97	-	20.29
1s	62.26	12.29	13.57
2s	82.55	20.29	15.10
3s	93.40	10.85	14.36

Table S5. Maximum cooling rate test results

**Time**	**Real-time temperature （°C）**	**Δt Temperature change value**	**v (°C /s)**
0s	93.82	-	18.26
1s	75.60	18.22	13.57
2s	57.33	18.26	15.10
3s	51.37	5.96	14.36

Table S6. Accuracy of Temperature Control

**Setpoints**	**45 (°C)**	**72 (°C)**	**95 (°C)**
Point 1	45.08	71.99	95.12
Point 2	45.16	72.12	94.99
Point 3	45.23	72.20	95.16
Point 4	45.06	72.27	95.03
Point 5	44.79	72.36	95.33
Mean temperature	45.07	72.19	95.13
Maximum of differences	0.07	0.19	0.13

Table S7. Temperature deviation between the PCR chamber and the reaction solution

**Setpoints (℃)**	**Chamber Temperature (°C)**	**Solution Temperature (°C)**	**Deviation （°C）**
50	49.97±0.12	49.47±0.15	0.5
57	57±0.17	58.2±0.10	1.2
94	94.10±0.10	92.33±0.06	1.77
